# Role of the Carbohydrate-Binding Sites of Griffithsin in the Prevention of DC-SIGN-Mediated Capture and Transmission of HIV-1

**DOI:** 10.1371/journal.pone.0064132

**Published:** 2013-05-31

**Authors:** Bart Hoorelbeke, Jie Xue, Patricia J. LiWang, Jan Balzarini

**Affiliations:** 1 Rega Institute for Medical Research, KU Leuven, Leuven, Belgium; 2 School of Natural Sciences, University of California Merced, Merced, California, United States of America; Institute of Human Virology, Baltimore, United States of America

## Abstract

**Background:**

The glycan-targeting C-type DC-SIGN lectin receptor is implicated in the transmission of the human immunodeficiency virus (HIV) by binding the virus and transferring the captured HIV-1 to CD4^+^ T lymphocytes. Carbohydrate binding agents (CBAs) have been reported to block HIV-1 infection. We have now investigated the potent mannose-specific anti-HIV CBA griffithsin (GRFT) on its ability to inhibit the capture of HIV-1 to DC-SIGN, its DC-SIGN-directed transmission to CD4^+^ T-lymphocytes and the role of the three carbohydrate-binding sites (CBS) of GRFT in these processes.

**Findings:**

GRFT inhibited HIV-1(III_B_) infection of CEM and HIV-1(NL4.3) infection of C8166 CD4^+^ T-lymphocytes at an EC_50_ of 0.059 and 0.444 nM, respectively. The single mutant CBS variants of GRFT (in which a key Asp in one of the CBS was mutated to Ala) were about ∼20 to 60-fold less potent to prevent HIV-1 infection and ∼20 to 90-fold less potent to inhibit syncytia formation in co-cultures of persistently HIV-1 infected HuT-78 and uninfected C8166 CD4^+^ T-lymphocytes. GRFT prevents DC-SIGN-mediated virus capture and HIV-1 transmission to CD4^+^ T-lymphocytes at an EC_50_ of 1.5 nM and 0.012 nM, respectively. Surface plasmon resonance (SPR) studies revealed that wild-type GRFT efficiently blocked the binding between DC-SIGN and immobilized gp120, whereas the point mutant CBS variants of GRFT were ∼10- to 15-fold less efficient. SPR-analysis also demonstrated that wild-type GRFT and its single mutant CBS variants have the capacity to expel bound gp120 from the gp120-DC-SIGN complex in a dose dependent manner, a property that was not observed for HHA, another mannose-specific potent anti-HIV-1 CBA.

**Conclusion:**

GRFT is inhibitory against HIV gp120 binding to DC-SIGN, efficiently prevents DC-SIGN-mediated transfer of HIV-1 to CD4^+^ T-lymphocytes and is able to expel gp120 from the gp120-DC-SIGN complex. Functionally intact CBS of GRFT are important for the optimal action of GRFT.

## Introduction

Several studies have shown that carbohydrate-binding agents (CBAs) act as efficient inhibitors of HIV entry [1]. They block virus entry by inhibiting the fusion of HIV virions with their target cells. CBAs are also able to prevent giant cell formation between HIV-infected and -uninfected CD4^+^ T-lymphocytes [2], [3]. DC-SIGN-mediated capture of HIV-1 and transmission of the DC-SIGN-captured virions to CD4^+^ T lymphocyte cells also represent an important pathway of infection [4] for which CBAs have also been shown to act on [1], [5]. CBAs are therefore promising candidates as potential HIV microbicides.

One of the most potent microbicide candidate drugs to date is griffithsin (GRFT). GRFT is a 121-amino acid dimeric lectin isolated from the red algae *Griffithsia* sp [6]. It has a very potent and broad spectrum anti-HIV-1 activity showing nano- to picomolar inhibitory activities against cell-free virus infection and cell-to-cell transfer of HIV [6], [7]. Recently it was also demonstrated that GRFT efficiently inhibits HIV-1 binding to the DC-SIGN receptor and subsequent migration of HIV-1 to CD4^+^ T-lymphocytes [8]. Furthermore GRFT exhibits a pronounced safety and efficacy profile since it effects only minimal changes in the induction of cytokines/chemokines in GRFT-exposed peripheral blood mononuclear cell (PBMC) cultures and it does not activate PBMC in cell culture [9]. Recombinant GRFT was shown to be non-toxic in the rabbit vaginal model and in human cervical explants [10]. Crystallization studies have demonstrated that GRFT occurs as a domain-swapped dimer, and the structural data have shown three putative carbohydrate-binding sites (CBS) on each monomer [11]–[13]. Xue et al [14] demonstrated that intact mannose-binding sites of GRFT are critical to preserve full anti-HIV-activity. However, the important negative impact on the antiviral potency of GRFT by single mutations in each of its CBS is not reflected in their virtually equally potent binding affinity towards gp120 as measured by ELISA and surface plasmon resonance (SPR) technology [14]. The latter findings are strongly suggestive for the requirement of cross-binding (avidity) of GRFT to the envelope gp120 in the native virus particles to afford potent antiviral efficacy [14]. It was recently also shown that binding of GRFT to HIV-1 gp120 affords a more pronounced exposure of the CD4 binding site [15] demonstrating that GRFT might have multiple effects on gp120, including altering its conformation.

Dendritic cells (DCs) are highly efficient antigen presenting cells which capture and degrade pathogens that enter the peripheral mucosal environment. They express a repertoire of pathogen-recognition receptors such as C-type lectins, which recognize molecular patterns expressed by pathogens [16]. DCs play an important role in the transmission of HIV-1 [16]–[18]. Immature DCs search the mucosal tissues for pathogens and are among the first cells to encounter HIV-1 during sexual transmission. Besides direct infection of DCs, the majority of HIV-1 virions are captured by DCs and transported to lymphoid tissues where upon transformation to mature DCs the virus is transferred to CD4^+^ T lymphocytes [19], [20]. By interaction of HIV-1 with DC-SIGN a fraction of the virus particles circumvents the natural role of DCs in the innate immune system by hijacking DCs for its migration from the mucosa to the secondary lymphoid organs instead of being taken up by DCs and getting degraded [8], [20]–[22]. The HIV-1 capture by dendritic cells is mediated by the terminal carbohydrate-recognition domain (CRD) of its C-type (Ca^2+^-dependent) DC-specific intercellular adhesion molecule 3 (ICAM-3)-grabbing nonintegrin (DC-SIGN) lectin [23]. Several studies revealed that the CRD of DC-SIGN recognizes α1,3- and α1,2-linked mannose oligomers [24] as well as fucosylated glycans [25].

Preventing HIV-1 interaction with DCs, and in particular DC-SIGN, represents an attractive approach to prevent HIV-1 transmission and infection of the host. We, therefore, investigated the potent CBA griffithsin and several mutant variants derived thereof on the capture of HIV-1 particles by Raji/DC-SIGN cells and the transmission of the captured virions to uninfected CD4^+^ T cells. We performed an extensive study on native GRFT and its mutant variants for their ability to inhibit the interaction between gp120 and DC-SIGN using surface plasmon resonance (SPR) technology. Our data shows that the potency of GRFT to inhibit DC-SIGN-mediated capture and transmission is markedly impaired when GRFT is mutated in one of its three carbohydrate-binding sites (CBS). This effect was much more pronounced for virus transmission than for virus capture.

## Materials and Methods

### Test compounds

Wild-type and mutant griffithsin (GRFT) were obtained as described before [14]. DC-SIGN-Fc was purchased from R&D Systems (Minneapolis, MN). Recombinant gp120 proteins from HIV-1(III_B_) and HIV-1(ADA) were produced in CHO cell cultures and obtained from ImmunoDiagnostics (Woburn, MA).

### Cells

CEM, C8166, HuT-78 and Sup-T1 cells were obtained from the American Type Culture Collection (Manassas, VA, USA). Persistently HIV-infected HuT-78/HIV cells were obtained upon cultivation of HuT-78 cell cultures exposed to HIV-1(III_B_) for 3 to 4 weeks. DC-SIGN-expressing Raji/DC-SIGN cells were constructed by Geijtenbeek *et al.* [26], [27] and kindly provided by L. Burleigh (Institut Pasteur, Paris, France). All cell lines were cultivated in RPMI-1640 medium (Invitrogen, Merelbeke, Belgium) supplemented with 10% fetal bovine serum (FBS) (BioWittaker Europe, Verviers, Belgium), 2 mM L-glutamine, 75 mM NaHCO_3_ and 20 µg/ml gentamicin (Invitrogen).

### Viruses

HIV-1(III_B_) was a kind gift from R.C. Gallo (Institute of Human Virology, University of Maryland, Baltimore, MD). HIV NL4.3 was obtained from ATCC (Rockville, MD).

### Antiretrovirus assays

C8166 and CEM cells (5×10^5^ cells per ml) were suspended in fresh culture medium and infected with HIV-1 (NL4.3) or HIV-1(III_B_) at 100 times the CCID_50_ (50% cell culture infective dose) per ml of cell suspension, of which 100 µl was mixed with 100 µl of the appropriate dilutions of the test compounds, and further incubated at 37°C. After 4 to 5 days, syncytia formation was recorded microscopically in the cell cultures, and the number of giant cells in the drug-treated cultures was estimated as a percentage of the number of giant cells present in the non-treated virus-infected cell cultures. The 50% effective concentration (EC_50_) corresponds to the compound concentrations required to prevent syncytium formation by 50% in the virus-infected CD4^+^ T-lymphocyte cell cultures [2], [3].

### Co-cultivation assay between uninfected T lymphocytes and persistently HIV-1-infected HuT-78 cells

Persistently HIV-1(III_B_)-infected HuT-78 cells (designated HuT-78/HIV-1) were washed to remove free virus from the culture medium, and 5×10^4^ cells (50 µl) were transferred to 96-well-microtiter plates. Next, a similar amount of CD4^+^ T-lymphocytes (50 µl) (i.e. Sup T1 or C8166) and appropriate concentrations of test compound (100 µl), were added to each well. After 2 days of co-culturing at 37°C, the EC_50_ values were quantified by microscopical inspection as based on the appearance of giant cells (syncytia) in the cocultures.

### Capture of HIV-1(III_B_) by Raji/DC-SIGN cells and co-cultivation of HIV-1-captured Raji/DC-SIGN cells with C8166 cells

The virus capture experiment was performed as originally described previously [28]. Briefly, exponentially growing B-lymphocyte DC-SIGN-expressing (Raji/DC-SIGN) cells were suspended in cell culture medium at 2×10^6^ cells/ml. Next, 100 µl of HIV-1 (NL4.3) (∼250,000 pg p24) was added to 500 µl of the Raji/DC-SIGN cell suspension in the presence of serial dilutions of the test compounds (400 µl). After 60 min of incubation, the cells were carefully washed to remove unbound virions and test compounds and resuspended in 1 ml of cell culture medium (10^6^ cells/ml). The captured HIV-1 (NL4.3 particles) by the Raji/DC-SIGN cells was quantified by a p24 Ag ELISA.

From a Raji/DC-SIGN cell suspension that contained captured HIV-1 (NL4.3), 200 µl (2×10^5^ cells) was mixed in a 48-well microplate with 800 µl uninfected C8166 cells (2×10^5^ cells). These cocultures were further incubated at 37°C, and syncytia formation was evaluated microscopically after ∼18 to 42 h. Viral p24 Ag quantification in the supernatants was performed by ELISA.

### Surface plasmon resonance (SPR) analysis

All interaction studies were performed with a Biacore T200 instrument (GE Healthcare, Uppsala, Sweden) at 25°C in HBS-P running buffer (10 mM HEPES, 150 mM NaCl and 0.05% surfactant P20; pH 7.4) supplemented with 10 mM Ca^2+^. Samples were injected for 2 minutes at a flow rate of 45 µl/min. To generate more information on the impact of GRFT on the interaction between gp120 and DC-SIGN two different SPR-based experiments were performed. In the first experimental set-up, the CM5 sensor chip was immobilized with recombinant HIV-1(III_B_) gp120 and HIV-1(ADA) gp120 in 10 mM sodium acetate, pH 4.0, using standard amine coupling chemistry at chip densities of 90 (∼0.8 fmol) and 130 RU (∼1.1 fmol), respectively. On this CM5 chip a competition assay with DC-SIGN and GRFT (both wild-type and mutant GRFT variants) for binding to immobilized gp120 was performed in which wild-type or mutated GRFT variants (each at 60–70x their [K_D_]) was administered for 2 minutes to immobilized gp120 and by the end of this time period, the initial GRFT concentration was kept but now in the additional presence of a fixed concentration of DC-SIGN (200 nM) and the binding signals were further recorded for an additional 120 sec after which the GRFT/DC-SIGN mixture in the analyte was replaced by elution buffer. In a second experimental set-up, a competition experiment was performed between HIV-1(III_B_) gp120 and GRFT (both wild-type and mutant GRFT) for binding to DC-SIGN, covalently immobilized on the carboxymethylated dextran matrix of a CM5 sensor chip (715 RU or 10.9 fmol bound DC-SIGN). In this assay DC-SIGN was first saturated with HIV-1(III_B_) gp120 (200 nM) during 2 minutes and by the end of this phase, the initial concentration of HIV-1(III_B_) gp120 was kept but now in the additional presence of varying concentrations of wild-type GRFT and mutated GRFT variants. The binding signals were further recorded for an additional 120 sec, after which the gp120/GRFT mixture in the analyte was replaced by elution buffer.

## Results

### Antiviral activity of GRFT and its mutant variants in HIV-infected cell cultures and in cocultures of persistently HIV-1-infected and uninfected CD4^+^ T-lymphocyte cells

The cytopathic effect of HIV on the CEM cell cultures was scored by microscopical reading and estimation of the number of virus-induced giant cells (syncytia). The antiviral effect of the CBAs was expressed as EC_50_ or compound concentration required to inhibit giant cell formation by 50%. The wild-type and mutant CBAs were investigated at concentrations that were considerably lower than their cytotoxicity in the cell cultures (>1 µM). GRFT and its single mutant variants inhibited the HIV-1-induced cytopathic effect in CEM and C8166 cell cultures (Table 1; Figures S1, S2, S3, S4, S5). The EC_50_ (50% effective concentration) values of wild-type GRFT for HIV-1(III_B_) and HIV-1(NL4.3) infection in CEM and C8166 cell cultures were 0.059 and 0.444 nM, respectively. The single mutant variants of GRFT (each D30A, D70A or D112A mutant destroying one of the three carbohydrate-binding sites in GRFT) were ∼25- to 70-fold less potent to prevent cell-free HIV-1 infection of CD4^+^ T-lymphocytes. The triple mutant (D30A/D70A/D112A) GRFT variant exhibited an approximately 400- to >900-fold diminished antiviral potency compared to wild-type GRFT with an EC_50_ of 52 nM and >185 nM for HIV-1(III_B_) and HIV-1(N.L4.3) infection in CEM and C8166 cell cultures, respectively. When the CBAs were exposed to a coculture of persistently HIV-1(III_B_)-infected HuT-78 cells and uninfected Sup-T1 or C8166 CD4^+^ T-lymphocytes, wild-type GRFT efficiently prevented syncytia formation in both type of cocultures at an EC_50_ of 1–2 nM (Table 1; Figures S1, S2, S3, S4, S5). The D30A and the D70A single mutants were also able to prevent syncytia formation in the cocultures but with a ∼40 to 90-fold decreased potency than wild-type GRFT. The mutant D112A GRFT variant was only ∼20-fold less active in these assays than wild-type GRFT. The triple GRFT mutant was unable to prevent syncytia formation between the persistently HIV-1-infected HuT-78/HIV-1 cells and the uninfected CD4^+^ T-lymphocytes at the highest tested concentrations (185 and 1852 nM) (Table 1; Figures S1, S2, S3, S4, S5).

**Table 1:** Anti-HIV-1 activity of GRFT and its mutant variants in different cell systems.

	HIV-1(III_B_) EC_50_ a (nM)	HIV-1(NL4.3) EC_50_ b (nM)	Hut-78/HIV-1(III_B_)+Sup-T1 EC_50_ c (nM)	HUT-78/HIV-1(III_B_)+C8166 EC_50_ d (nM)
GRFT WT	0.059±0.0	0.444±0.052	1.0±0.67	2.1±0.2
GRFT D30A	3.7±0.31	27±14	37±21	126±8
GRFT D70A	3.63±1.2	30±10	48±13	193±56
GRFT D112A	2.6±0.29	9.3±6.3	20±7.8	52±2.6
GRFT D30A/D70A/D112A	52±18	>185	>185	>1852

a EC_50_ required to inhibit virus-induced cytopathicity in CEM (HIV-1 III_B_) cell cultures by 50%.

b EC_50_ required to inhibit virus (HIV-1 NL4.3)-induced cytopathicity in C8166 cell cultures by 50%.

c 50%-Effective concentration or compound concentration required to inhibit syncytia formation between persistently HIV-1(III_B_)-infected HuT-78/HIV-1 cells and uninfected CD4^+^ T-lymphocyte Sup T1 cells by 50%.

d 50%-Effective concentration or compound concentration required to inhibit syncytia formation between persistently HIV-1(III_B_)-infected HuT-78/HIV-1 cells and uninfected CD4^+^ T-lymphocyte C8166 cells by 50%.

The data from which the EC_50_'s were derived are shown in Figures S1, S2, S3, S4, S5 and are the mean of at least 2 to 3 independent experiments.

### Effect of GRFT and its mutant variants on the capture of HIV-1 by Raji/DC-SIGN cells and transmission of captured HIV-1 to uninfected CD4+ T-lymphocyte C8166 cells

**Figure 1:**
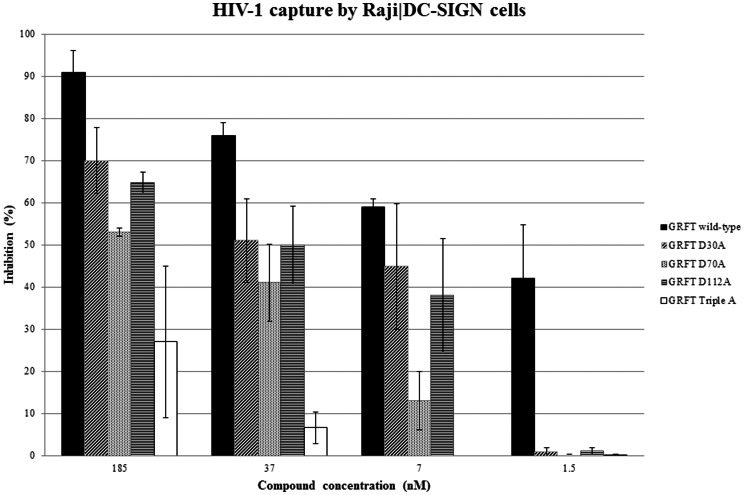
Inhibitory activity of GRFT and its mutant variants against DC-SIGN-mediated capture of HIV-1(NL4.3) by Raji/DC-SIGN. The control cultures contained DC-SIGN-captured virus at 3.77±0.37 ng p24/10^6^ cells (data represent the mean of 2 independent experiments).

**Figure 2:**
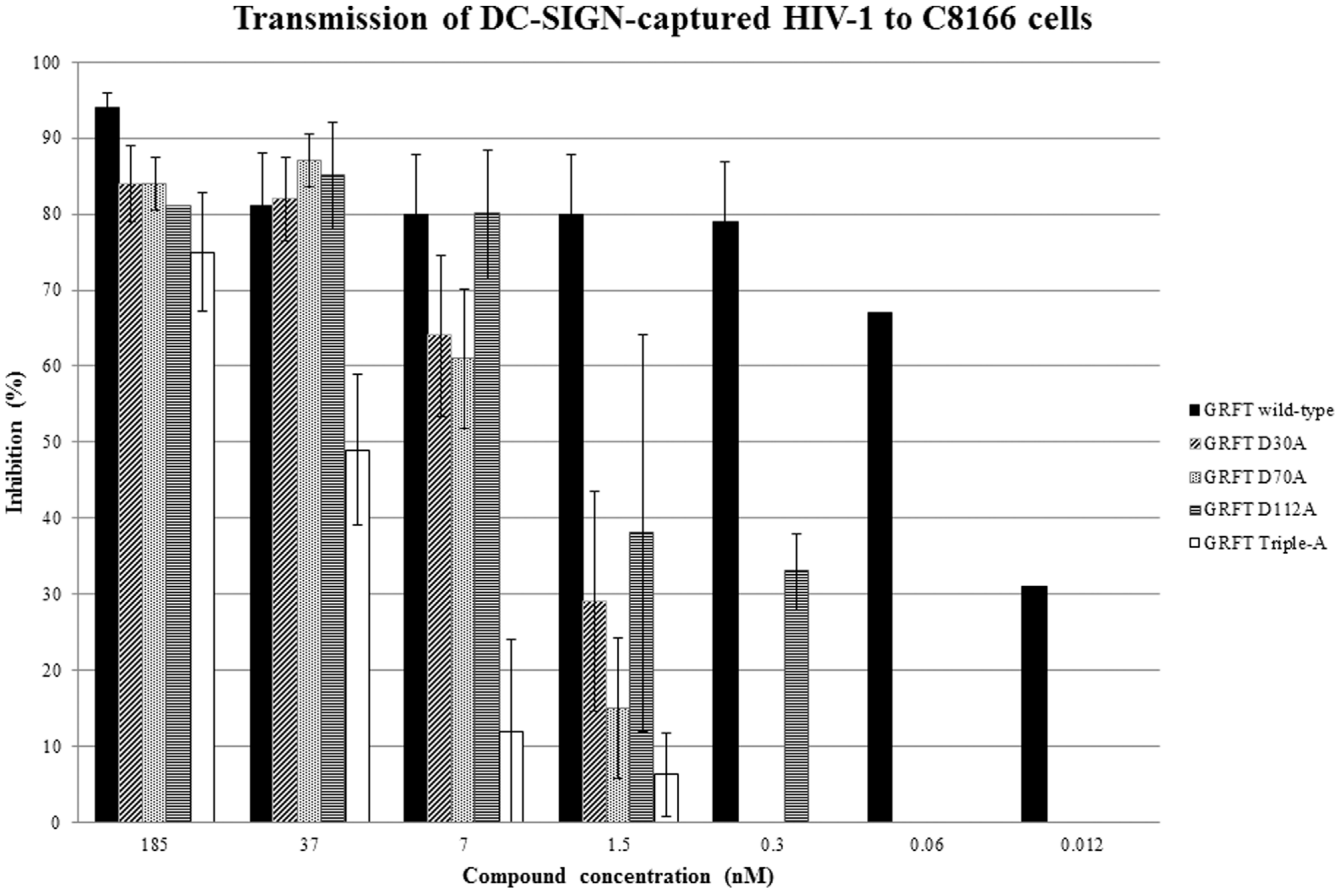
Inhibitory activity of GRFT and its mutant variants against DC-SIGN-mediated virus transmission of HIV-1-captured virions to CD4^+^ T-lymphocyte cells. The HIV-1-captured Raji/DC-SIGN cultures used for virus transmission in the presence or absence of the wild-type and mutant GRFT variants contained following amounts of captured HIV-1: 5.50±2.09 ng p24/10^6^ cells (for the WT GRFT experiments), 4.30±0.15 ng p24/10^6^ cells (for the mutant D30A GRFT experiments), 3.66±0.59 ng p24/10^6^ cells (for the mutant D70A GRFT experiments), 4.04±0.72 ng p24/10^6^ cells (for the mutant D112A GRFT experiments), and 3.78±0.77 ng p24/10^6^ cells (for the mutant Triple A GRFT experiments). Data represent the mean of three independent experiments.

GRFT has been examined for its potential to prevent HIV-1(NL4.3) capture by DC-SIGN-expressing Raji cells and to decrease the transmission of DC-SIGN-captured virions to uninfected CD4^+^ T-lymphocyte C8166 cells. In a first set of experiments, HIV-1(NL4.3) was exposed to different concentrations of GRFT and its mutant variants during 30 min before the virus was added to the DC-SIGN-expressing Raji cell cultures. One hour later, free virus particles and the test compounds were carefully removed from the cell cultures by several washing steps. P24 Ag ELISA analysis revealed that GRFT dose-dependently inhibited HIV-1(NL4.3) capture by Raji/DC-SIGN cells and almost completely blocked the capture at the highest concentration tested, i.e. 185 nM (Fig. 1). In contrast, the inhibition of the capture of HIV-1 virions by single GRFT mutants was less pronounced than wild-type GRFT. At the lowest concentrations (i.e. 1.5 nM), none of the mutant GRFT variants prevented virus capture whereas wild-type GRFT was still able to inhibit virus capture by 40% at this concentration (Fig. 1). The triple mutant GRFT variant was clearly much less effective blocking virus capture by DC-SIGN-expressing cells than the single mutant variants. At 7 to 37 nM concentrations, capture was hardly (∼6% at 37 nM) or not at al (0% at 7 nM) prevented (Fig. 1). In a second set of experiments, virus-captured Raji/DC-SIGN cells were co-cultured with CD4+ T-lymphocytes C8166 cells in the presence of wild-type and the mutant GRFT variants, and giant cell formation was recorded microscopically within 24 to 48 hours post cocultivation. The cytopathic effect (appearing after the transmission of HIV-1 from the virus-captured Raji/DC-SIGN cells to the C8166 cells and subsequent virus replication in the C8166 cell cultures) was recorded as a parameter of efficiency of virus transmission. Wild-type GRFT inhibited HIV-1 transmission almost completely (>95%) at 185 nM but was still very efficient at subnanomolar concentrations (Fig. 2). The mutant GRFT variants inhibited HIV-1 transmission at similar orders of magnitude in the presence of 7 to 185 nM concentrations as wild-type GRFT. However, at lower (i.e. 5 nM) concentrations of the test compounds, wild-type GRFT still blocked virion transmission by ∼80%, whereas the mutant GRFT variants lost considerable potency, and with the exception of the mutant D112A GRFT variant, the other mutant GRFT variants completely lost their potential to block virus transmission at concentrations of 0.3 nM and lower. The triple mutant GRFT variant proved by far inferior to the single GRFT mutants regarding its potential to block DC-SIGN-captured virus transmission (Fig. 2). The data also revealed that WT GRFT was approximately 100-fold more effective at inhibiting HIV-1 transmission compared to its capability to prevent the capture of virus particles by Raji/DC-SIGN cells (IC_50_s derived from Fig. 1 and Fig. 2: ∼5 nM and 0.05 nM, respectively) and that the single mutant GRFT variants were 20- to 100-fold less effective than wild-type GRFT against both virus capture and transmission.

### Competition of GRFT and DC-SIGN for binding to immobilized recombinant HIV-1 gp120

To further investigate and clarify the molecular basis of the above-described observations obtained in cell culture, we studied by SPR technology the binding of recombinant DC-SIGN with immobilized III_B_ gp120 (X4) when wild-type GRFT or one of the single mutant variants is already bound to HIV-1(III_B_) gp120. For this purpose, 5 nM wild-type GRFT; 10 nM mutant D30A GRFT; 20 nM mutant D70A GRFT and 10 nM mutant D112A GRFT (each concentration representing ∼60–70-fold their corresponding K_D_ value for gp120 binding) were administered for 2 minutes to immobilized gp120 and immediately at the end of the association phase, 200 nM DC-SIGN was added. Similar experiments were performed on immobilized HIV-1 ADA gp120 (R5). In these experimental set-ups we wanted to mimic the *in vivo* capture step of HIV-1 (gp120) virions by the DC-SIGN receptor in the presence of the CBAs. When 5 nM wild-type GRFT was pre-exposed to gp120, only 4.5 to 5.9% of DC-SIGN could bind to gp120 (Table 2; Figure S6). In the presence of the mutant D30A and D112A GRFT variants DC-SIGN binding signals were 52% and 37% of control (absence of GRFT) for immobilized HIV-1(III_B_) gp120 and HIV-1 ADA gp120, respectively. The mutant D70A GRFT variant was least efficient in its inhibitory potential against the binding of DC-SIGN to immobilized gp120 (80 and 50% DC-SIGN binding on mutant D70A GRFT-pre-bound gp120) (Table 2; Figure S6). The data revealed that wild-type GRFT can efficiently inhibit the binding between gp120 and DC-SIGN in contrast to the point mutant variants which were estimated to be ∼10 to 15-fold less efficient, as estimated from their binding curves (data not shown).

**Table 2:** Binding efficiency of DC-SIGN to immobilized HIV-1 gp120 III_B_ and HIV-1 gp120 ADA that were pre-exposed to GRFT and its mutant variants.

CBA^a^	% DC-SIGN binding on CBA exposed gp120 III_B_	% DC-SIGN binding on CBA exposed gp120 ADA
GRFT WT (5 nM)	5.9±3.5	4.5±3.9
GRFT D30A (10 nM)	52±13	37±1
GRFT D70A (20 nM)	79±26	50±2
GRFT D112A (10 nM)	54±0.2	40±3

a Concentration used in the study represents a 60- to 70-fold K_D_ concentration of the particular CBA against HIV-1 gp120. Data are the mean of 2 independent experiments. The SPR data are shown in Figure S6.

### Competition of GRFT and gp120 for binding to immobilized recombinant DC-SIGN

**Figure 3:**
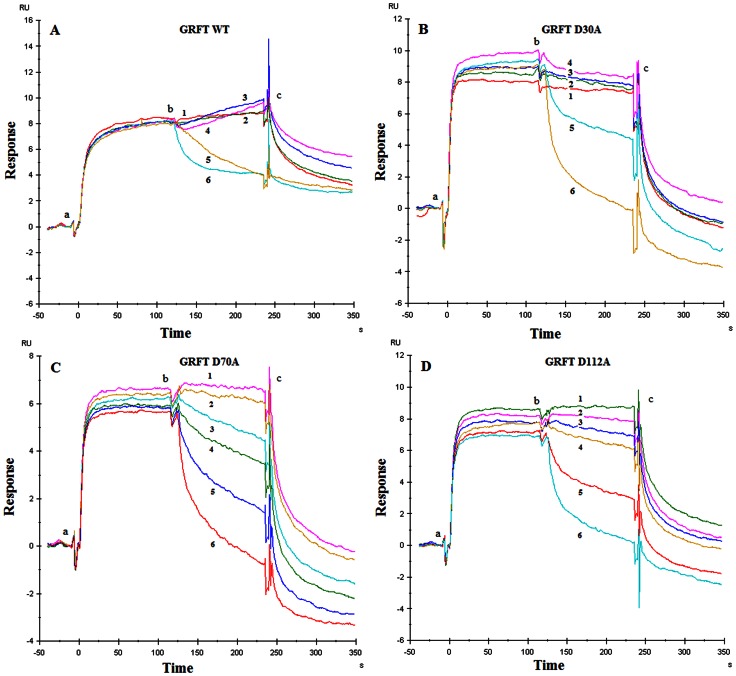
SPR-based competition experiment between CBA and gp120 III_B_ for binding to immobilized DC-SIGN (chip density 715 RU ∼10.9 fmol). Panel A: Effect of GRFT exposure on the HIV-1(III_B_) gp120 complex with DC-SIGN. 200 nM gp120 was injected (time point a), followed after 2 min by an additional injection (time point b) of varying concentrations of GRFT (0.1 nM [1], 1 nM [2], 10 nM [3], 25 nM [4], 100 nM [5] and 250 nM [6]). Similar experiments are depicted in Panels B and D but for varying concentrations of mutant D30A GRFT variant and mutant D112A GRFT variant (0.2 nM [1], 2 nM [2], 20 nM [3], 50 nM [4], 200 nM [5] and 500 nM [6]), respectively. In Panel C the concentrations of mutant D70A GRFT variant were 0.4 nM [1], 4 nM [2], 40 nM [3], 100 nM [4], 400 nM [5] and 1000 nM [6]. The curves show a representative example out of two independent experiments.

**Figure 4:**
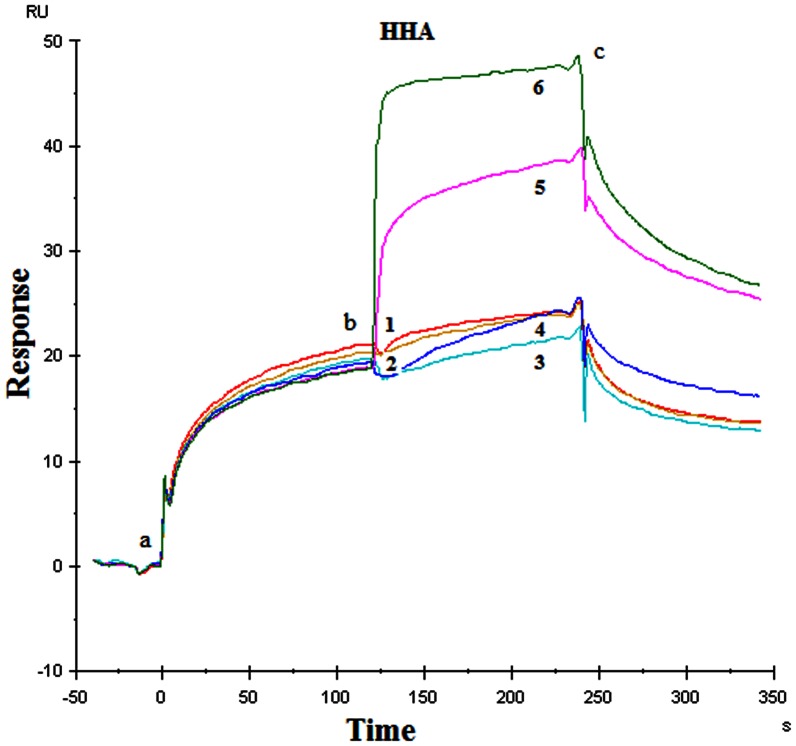
SPR-based competition experiment between CBA and gp120 IIIB for binding to immobilized DC-SIGN (chip density 1050 RU ∼16 fmol). Effect of HHA exposure on the HIV-1(III_B_) gp120 complex with DC-SIGN. 200 nM gp120 was injected (time point a), followed after 2 min by an additional injection (time point b) of varying concentrations of HHA (0.4 nM [1], 4 nM [2], 40 nM [3], 100 nM [4], 400 nM [5] and 1000 nM [6]).

Similar competition experiments were performed as described above but now DC-SIGN was covalently immobilized on the sensor chip instead of HIV-1 gp120 (Fig. 3, Panels A–D). 200 nM HIV-1(III_B_) gp120 was injected at time point (a) and kept at time point (b) at which, additionally, varying concentrations of wild-type GRFT and its mutant variants were injected for another 2 min (time point (c)) (Fig. 3, Panel A: wild-type GRFT; Fig. 3, Panel B: mutant D30A GRFT; Fig. 3, Panel C: mutant D70A GRFT; and Fig. 3, Panel D: mutant D112A GRFT). With this SPR-based experiment we aimed to investigate whether, when DC-SIGN is already bound to gp120 (which mimics the virus capture step by DC-SIGN), GRFT is still able to additionally bind to gp120 at glycan sites not covered by DC-SIGN and/or whether GRFT can destroy the DC-SIGN-gp120 complex. It was generally observed that, when gp120 is already bound to DC-SIGN (time point (b) in Fig. 3), wild-type GRFT and its single mutant variants are capable to interfere with the DC-SIGN-gp120 interaction as they all have the capacity to remove bound gp120 from the immobilized DC-SIGN in a dose-dependent manner (resulting in decreased RU values). The latter effect was not observed for *Hippeastrum* hybrid agglutinin (HHA), another potent mannose-specific anti-HIV CBA when included in similar assays at an HHA concentration range between 0.4 and 100 nM (Fig. 4). However, in contrast with the mutant GRFT variants, the lower concentrations of wild-type GRFT (ranging between 0.1 to 25 nM) rather resulted in an increase in RU (Figure 2 Panel A, time points between b and c), presumably due to its binding to the DC-SIGN-gp120 complex. This phenomenon was also observed for HHA when administered at 1,000 and 400 nM to the DC-SIGN-gp120 complex (Fig. 4).

## Discussion

Carbohydrate-binding agents (CBAs) such as the prokaryotic cyanovirin (CV-N) and actinohivin (AH) and several plant lectins have been shown to inhibit binding of HIV to DC-SIGN^+^ cells and to prevent transmission of DC-SIGN-captured HIV to CD4^+^ T-lymphocyte cells [5], [29], [30]. Here, we investigated the capability of griffithsin (GRFT) to interact with the HIV capture/transmission process. Three single mutant GRFT variants each containing a single mutation at one of the three carbohydrate-binding sites of GRFT (D30A, D70A and D112A) (resulting in the selective deletion of each CBS as previously described [14]) were also included in this study to obtain better insight in the role of the individual CBS of GRFT in its inhibitory activity against virus infection, capture and transmission. We showed that wild-type GRFT potently inhibited cell-free HIV infection (EC_50_: 0.05–0.4 nM) and also efficiently blocked cell-to-cell virus transmission (EC_50_: 1–2 nM) in the co-cultivation assay using HuT-78/HIV-1 and Sup-T1 or C8166 cells (Table 1). We also demonstrated that wild-type GRFT efficiently prevents the DC-SIGN-directed capture of HIV-1 at an EC_50_ of ∼5 nM (Fig. 1) and the transmission of DC-SIGN-captured HIV-1 to uninfected CD4^+^ T-lymphocytes at even a 100-fold lower concentration (EC_50_ of ∼0.05 nM) (Fig. 2). Our capture/transmission findings on wild-type GRFT are also in agreement with those of Alexandre et al. who recently demonstrated that several CBAs including GRFT, cyanovirin and scytovirin inhibited both HIV-1 binding to DC-SIGN and DC-SIGN-mediated HIV-1 infection of CD4^+^ T-lymphocytes (8). They also showed that inhibition of DC-SIGN-directed virus capture by these lectins is less efficient than their inhibitory potential against virus transmission, an observation which is again in line with our findings. The picomolar to low nanomolar effective concentrations sufficient to concomitantly block or prevent HIV-1 infection and virus capture and virus transmission is without precedent among the CBAs. These agents, such as GRFT, represent the only entities that concomitantly can interact with the different multiple pathways HIV-1 uses for its efficient infection/transmission. These properties are important in view of the design and development of efficient microbicide candidate drugs.

The single mutant variants of GRFT were markedly (20- to 90-fold) less efficient in preventing cell-free HIV-1 infection and blocking cell-cell HIV-1 transfer in our assay systems. The antiviral data for the mutant GRFT variants obtained in C8166 cell cultures were comparable with those obtained in CEM cell cultures and were also in line with the findings very recently reported by Xue et al. [14].

The mutant GRFT variants inhibit DC-SIGN-directed HIV-1 binding at an IC_50_ of ∼40 nM, which is ∼8-fold less effective than wild-type GRFT, but still more potent than other lectins such as the plant lectin HHA (IC_50_: ∼45 nM) [5] and the prokaryotic lectin actinohivin (IC_50_: ∼224 nM) [30]. The triple mutant GRFT variant more dramatically lost its capacity to prevent HIV-1 capture by DC-SIGN (IC_50_: >185 nM). In the in vitro assays using DC-SIGN^+^ Raji cells the mutant GRFT variants showed a ∼100-fold decrease in potency to inhibit the transmission of DC-SIGN-bound HIV-1 to C8166 cells than wild-type GRFT. The clearly lower inhibitory potency of wild-type GRFT and its mutant variants against DC-SIGN-directed virus capture *versus* cell-free virus infection of CD4^+^ T-lymphocytes and DC-SIGN-directed transmission of HIV to CD4^+^ T-lymphocytes may suggest that DC-SIGN recognizes, at least partially, different glycan epitopes on gp120 than GRFT and/or recognizes some glycan epitopes more efficiently than GRFT and therefore, relatively high GRFT concentrations are needed to prevent DC-SIGN-mediated HIV-1 capture. In contrast, the GRFT concentration needed to substantially prevent T-lymphocyte infection and DC-SIGN-captured HIV transmission to CD4^+^ T-lymphocytes is markedly lower. These findings are in agreement with the SPR data obtained for inhibition of DC-SIGN binding to gp120 that was pre-exposed to GRFT.

Previously, the binding constants of wild-type GRFT and its mutant variants for HIV-1 gp120 have been determined by surface plasmon resonance technology [14]. It was found that the single mutations did not have an important impact on the affinity constant (K_D_) of the lectin, and ranged between 72.7 and 244.9 pM (K_D_: 72.7 pM for wild-type GRFT, 155.6 pM for mutant D30A GRFT, 244.9 pM for mutant D70A GRFT and 141.8 pM for mutant D112A GRFT. In our experiments GRFT concentrations of ∼70-fold the K_D_ value of GRFT for gp120 binding were needed to prevent DC-SIGN binding to HIV-1 by ∼95% (Table 2). However, it was interesting to note that the single mutant GRFT variants, although exposed to gp120 at equal K_D_-fold concentrations as wild-type GRFT, were at least ∼10- to 20-fold less effective than wild-type GRFT to prevent DC-SIGN binding to the gp120/GRFT variant complexes (Table 2). These findings may be explained by superior cross-linking/avidity properties of wild-type GRFT *versus* the mutant GRFT variants, and therefore, bound wild-type GRFT to gp120 may more efficiently prevent (hinder) DC-SIGN for additional binding to the gp120/GRFT complex than its mutant variants. This phenomenon and the importance of the capability of CBAs such as GRFT to concomitantly cross-link several glycans to exert efficient antiviral activity is also evident from our findings that GRFT and its single mutant variants show virtually similar low K_D_ values (binding affinity) for gp120, but have strikingly different antiviral potencies (wild-type GRFT is by far superior to the mutant GRFT variants in its antiviral efficacy) ([14], and data in this study). The importance of cross-linking glycans on gp120 by CBAs has not only be recently shown for GRFT ([14] and this study), but also for other CBAs such as the prokaryotic cyanovirin [31], the plant lectins of *Sambucus sieboldiana*, Soybean, *Wisteria floribunda* and *Vicia villosa* [32], [33], the snail lectin of *Helix pomatia* [33] and the mammalian galectins [34].

Experiments were also carried out in which DC-SIGN was immobilized on the sensor chip and exposed to gp120 before GRFT and its mutant variants were administered. Such experimental set-up was meant to investigate whether GRFT was able to interfere with the DC-SIGN/gp120 complex once already formed. Two phenomena were observed. (i) GRFT (and its mutants) were able to expell (remove) gp120 from the DC-SIGN/gp120 complex in a dose-dependent manner, as evident by a dose-dependent decrease of the RU amplitude in the SPR experiments at different GRFT concentrations. This means that GRFT is able to directly (and strongly) compete with the glycan binding sites of DC-SIGN and/or that GRFT is able to bind to glycan sites distant from the DC-SIGN-binding glycans but allosterically lowering the affinity of DC-SIGN for the other glycans on gp120. In fact, it has been shown that GRFT binding to gp120 may significantly increase the exposure of the CD4 binding site on gp120 by affecting (changing) the conformation of gp120 [15], and thus it cannot be excluded that such conformational changes might also affect the affinity of DC-SIGN to the gp120 glycan in the presence of GRFT.

When the mutant GRFT variants were compared at a fixed concentration of 20 nM (Fig. 3, Panel B curve 3; Panel C in between curve 2 and 3; and Panel D curve 3), a decrease of gp120 binding to DC-SIGN was detected to the extent of 10 to 15% whereas wild-type GRFT showed an increased binding signal at this concentration (see also Table 3 in which the amplitudes are given for the DC-SIGN/gp120/GRFT binding signal as calculated from the concentration curves of Fig. 3). Most likely, the increased binding signals at the lower concentrations of wild-type GRFT are due to the strong binding capacity of wild-type GRFT to gp120. We hypothesize that at these low concentrations wild-type GRFT still binds to gp120 through remaining free glycans on gp120 that were not covered by DC-SIGN and thus results in a higher total mass bound to the sensor chip. As a result, the SPR signal is not decreasing but rather (slightly) increasing due to the additional binding of GRFT to remaining free glycans on the gp120-DC-SIGN complex. At higher GRFT concentrations, however, GRFT out-competes gp120 in its binding to DC-SIGN, resulting in a release of gp120 (bound to GRFT) from the DC-SIGN-gp120 complex, and thus resulting in a dose-dependently decreased SPR signal. This phenomenon is not observed for the α1,3/α1,6-mannose-specific HHA (Fig. 4). In fact, at 400 and 1,000 nM HHA, additional binding of this CBA to the DC-SIGN-gp120 complex was observed. Thus, the gp120-expelling activity of GRFT does not seem to be a general property of other related CBAs which may add to the potential of GRFT as an attractive microbicide candidate CBA.

**Table 3:** Increased (positive sign) or decreased (negative sign) amplitude (RU) upon GRFT exposure to the DC-SIGN-gp120 complex.

[CBA]	GRFT WT	GRFT D30A	GRFT D70A	GRFT D112A
1x [K_D_]	5	−9	0	0
10x [K_D_]	10	−12	−6	−5
100x [K_D_]	23	−15	−29	−12
250x [K_D_]	17	−21	−42	−22
1000x [K_D_]	−44	−61	−76	−60
2500x [K_D_]	−53	−114	−112	−97

In conclusion, GRFT was found to be a potent inhibitor of HIV infection, DC-SIGN-directed virus capture and transmission of DC-SIGN-captured virus to CD4^+^ T-lymphocytes. Functionally intact carbohydrate-binding sites on the GRFT molecule are important for its optimal activity. SPR studies revealed that GRFT is able to expel gp120 from the DC-SIGN/gp120 complex when exposed to the DC-SIGN/gp120 complex at middle to higher nanomolar concentrations (25–250 nM), an observation that is important for its potential clinical application as a microbicide drug.

## Supporting Information

Figure S1 **Inhibition of virus-induced cytopathicity (syncytia formation) in HIV-1/NL4.3-infected C8166 cell cultures, HIV-1(III_B_)-infected CEM cell cultures, cocultures of HUT-78/HIV-1(III_B_) and C8166 cells, and cocultures of HuT-78/HIV-1(III_B_) and Sup T1 cells in the presence of a variety of WT GRFT concentrations.** Data represent the mean of at least two to three independent experiments.
(TIF)

Figure S2 **Inhibition of virus-induced cytopathicity (syncytia formation) in HIV-1/NL4.3-infected C8166 cell cultures, HIV-1(III_B_)-infected CEM cell cultures, cocultures of HUT-78/HIV-1(III_B_) and C8166 cells, and cocultures of HuT-78/HIV-1(III_B_) and Sup T1 cells in the presence of a variety of mutant D30A GRFT concentrations.** Data represent the mean of at least two to three independent experiments.
(TIF)

Figure S3 **Inhibition of virus-induced cytopathicity (syncytia formation) in HIV-1/NL4.3-infected C8166 cell cultures, HIV-1(III_B_)-infected CEM cell cultures, cocultures of HUT-78/HIV-1(III_B_) and C8166 cells, and cocultures of HuT-78/HIV-1(III_B_) and Sup T1 cells in the presence of a variety of mutant D70A GRFT concentrations.** Data represent the mean of at least two to three independent experiments.
(TIF)

Figure S4 **Inhibition of virus-induced cytopathicity (syncytia formation) in HIV-1/NL4.3-infected C8166 cell cultures, HIV-1(III_B_)-infected CEM cell cultures, cocultures of HUT-78/HIV-1(III_B_) and C8166 cells, and cocultures of HuT-78/HIV-1(III_B_) and Sup T1 cells in the presence of a variety of mutant D112A GRFT concentrations.** Data represent the mean of at least two to three independent experiments.
(TIF)

Figure S5 **Inhibition of virus-induced cytopathicity (syncytia formation) in HIV-1/NL4.3-infected C8166 cell cultures, HIV-1(III_B_)-infected CEM cell cultures, cocultures of HUT-78/HIV-1(III_B_) and C8166 cells, and cocultures of HuT-78/HIV-1(III_B_) and Sup T1 cells in the presence of a variety of mutant Triple A GRFT concentrations.** Data represent the mean of at least two to three independent experiments.
(TIF)

Figure S6 **SPR-based competition experiment between CBAs and DC-SIGN for binding to immobilized gp120 ADA (chip density 130 RU ∼1.1 fmol) (Panels A–E) and gp120 IIIB (chip density 90 RU ∼0.8 fmol) (Panels F–J).** Panel A: Injection of 200 nM DC-SIGN on gp120 ADA. Panel B: Effect of DC-SIGN exposure to HIV-1(ADA) gp120 that had been preexposed by WT GRFT. 5 nM WT GRFT was injected (time point a; red and green curves), followed after 2 min by an additional injection (time point b) of 5 nM WT GRFT (red curve) or WT GRFT +200 nM DC-SIGN (green curve). In Panel C till E similar experiments were carried out but 5 nM WT GRFT is replaced by 10 nM D30A GRFT; 20 nM D70A GRFT or 10 nM D112A GRFT, respectively. Data of similar experiments are shown in Panels F till J, using gp120_IIIB_ as the covalently linked analyte. The curves show a representative example out of two independent experiments.
(TIF)
