# Supplementary figures and images for: Role of the Carbohydrate-Binding Sites of Griffithsin in the Prevention of DC-SIGN-Mediated Capture and Transmission of HIV-1
Source: PLoS One. 2013 May 31;8(5):e64132. doi: 10.1371/journal.pone.0064132 (PMC3669349; doi:10.1371/journal.pone.0064132)

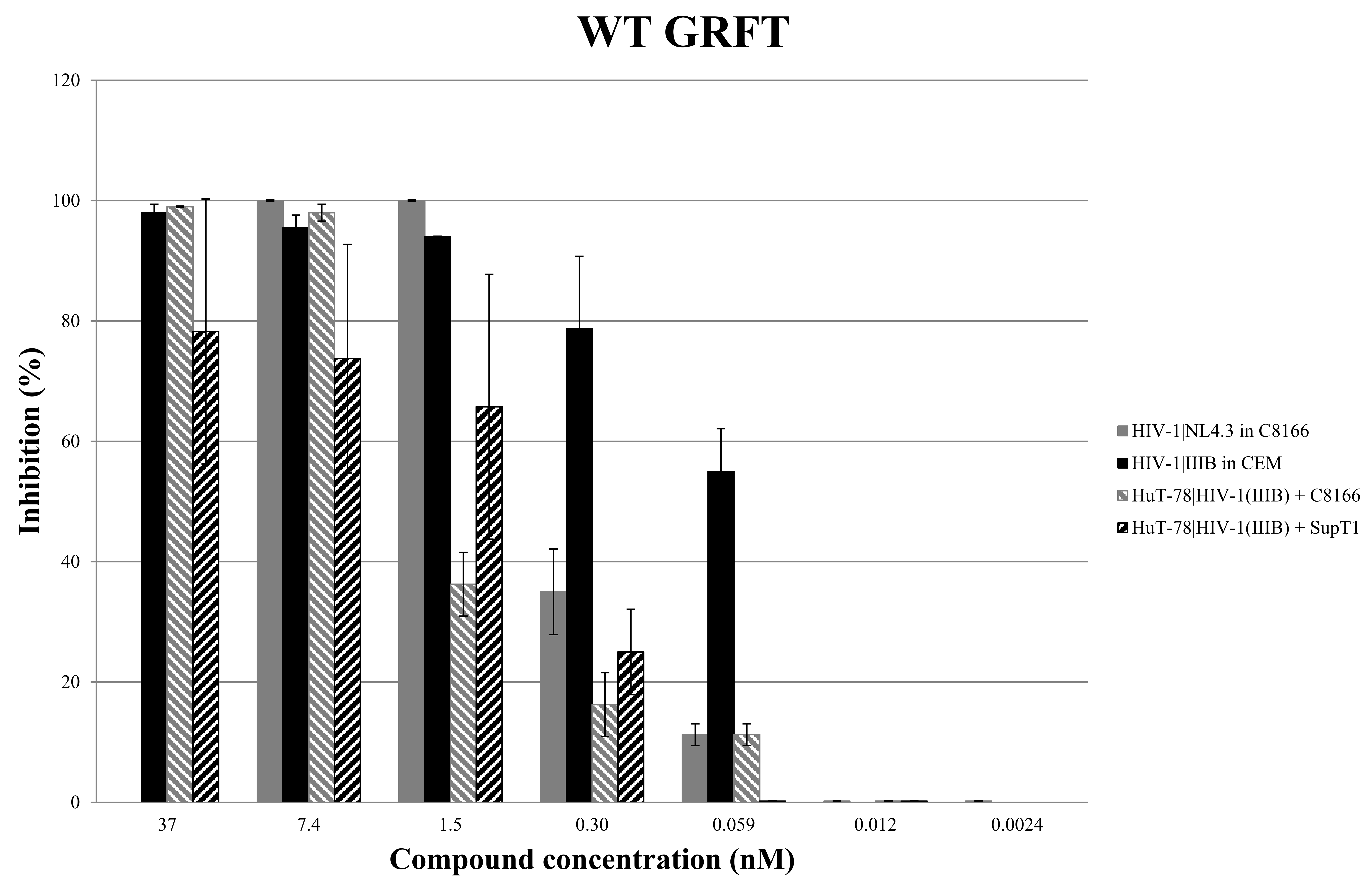

Supplement: Figure S1 — Inhibition of virus-induced cytopathicity (syncytia formation) in HIV-1/NL4.3-infected C8166 cell cultures, HIV-1(IIIB)-infected CEM cell cultures, cocultures of HUT-78/HIV-1(IIIB) and C8166 cells, and cocultures of HuT-78/HIV-1(IIIB) and Sup T1 cells in the presence of a variety of WT GRFT concentrations. Data represent the mean of at least two to three independent experiments. (TIF) [file pone.0064132.s001.tif]

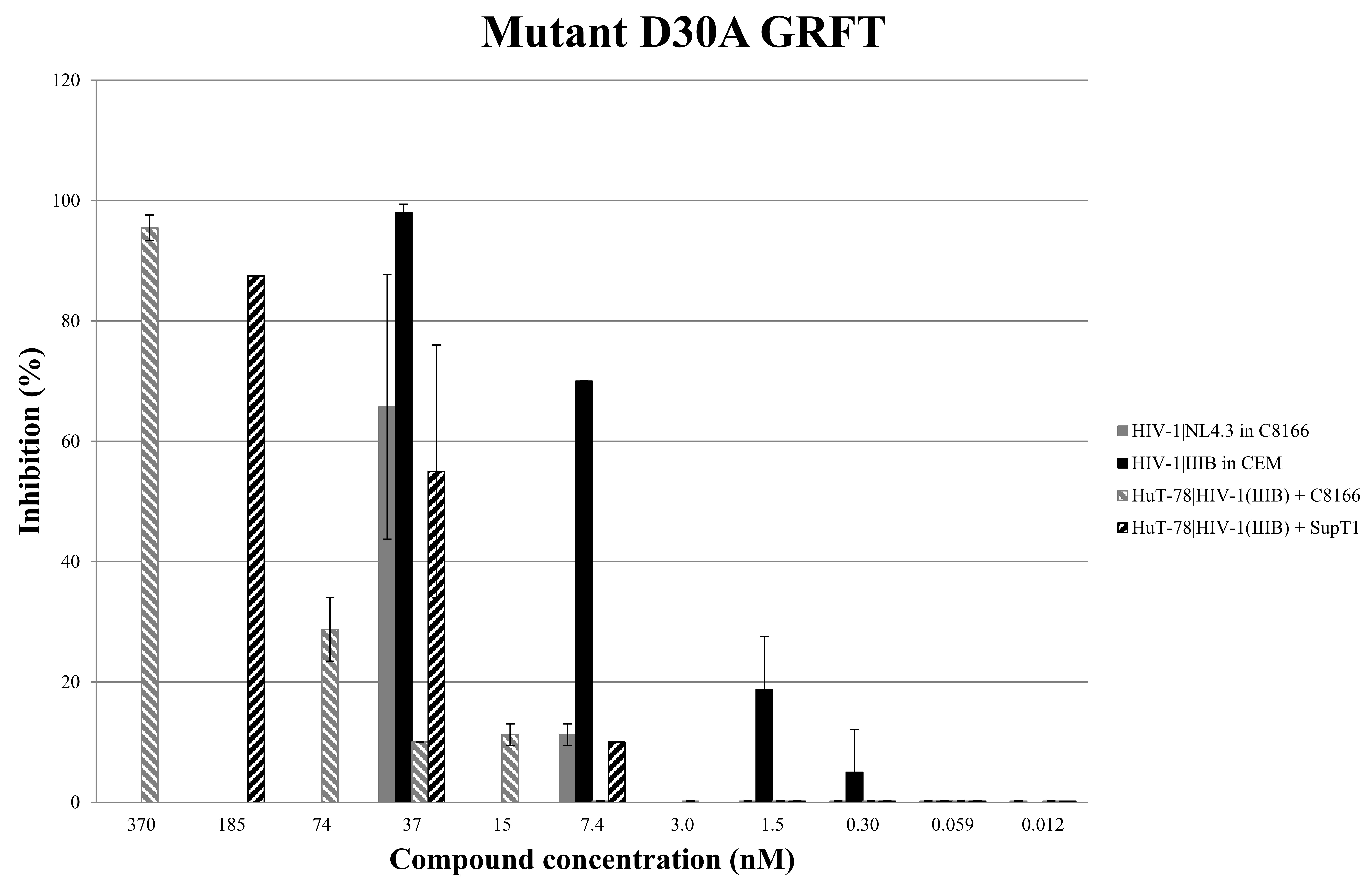

Supplement: Figure S2 — Inhibition of virus-induced cytopathicity (syncytia formation) in HIV-1/NL4.3-infected C8166 cell cultures, HIV-1(IIIB)-infected CEM cell cultures, cocultures of HUT-78/HIV-1(IIIB) and C8166 cells, and cocultures of HuT-78/HIV-1(IIIB) and Sup T1 cells in the presence of a variety of mutant D30A GRFT concentrations. Data represent the mean of at least two to three independent experiments. (TIF) [file pone.0064132.s002.tif]

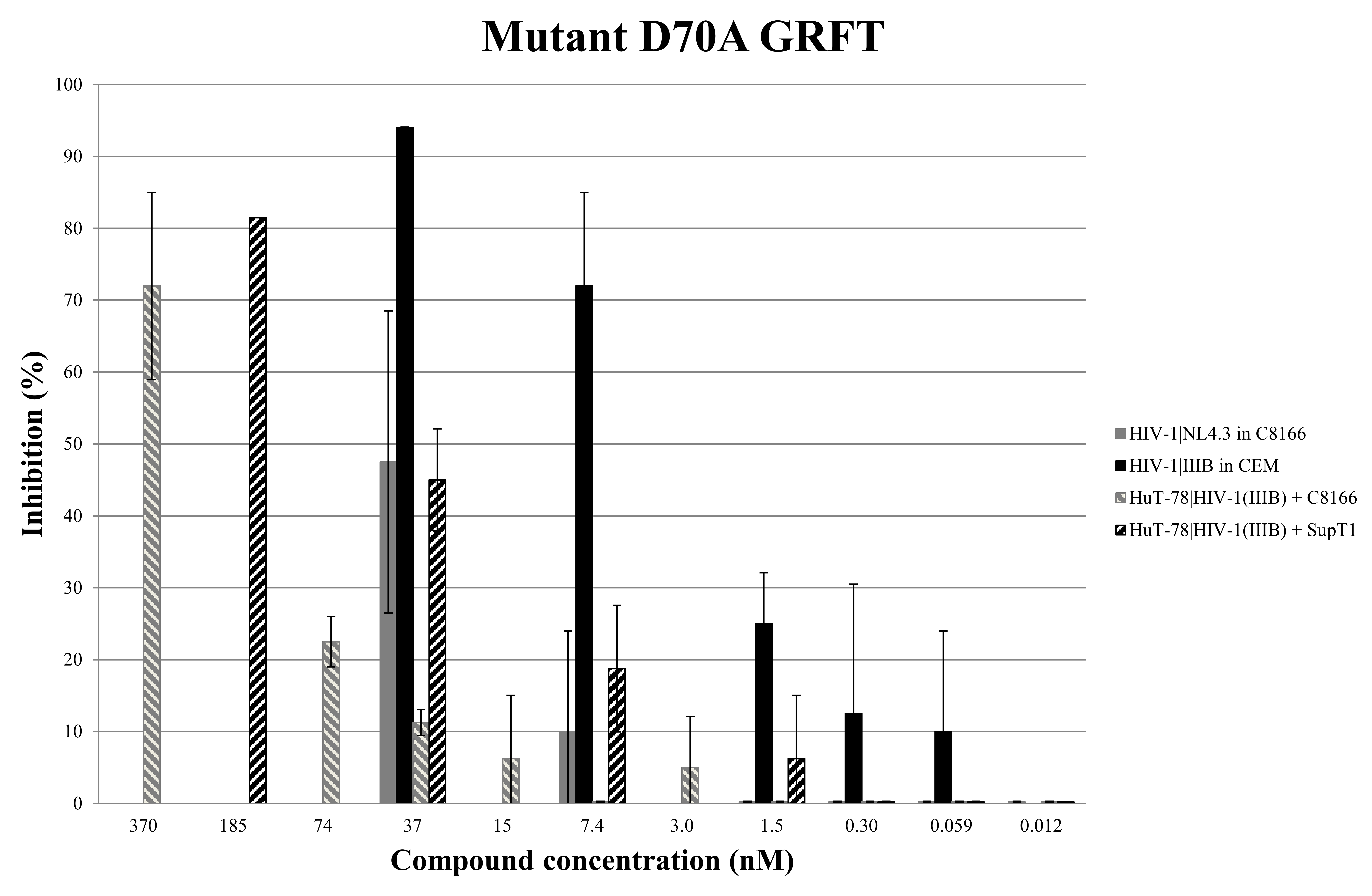

Supplement: Figure S3 — Inhibition of virus-induced cytopathicity (syncytia formation) in HIV-1/NL4.3-infected C8166 cell cultures, HIV-1(IIIB)-infected CEM cell cultures, cocultures of HUT-78/HIV-1(IIIB) and C8166 cells, and cocultures of HuT-78/HIV-1(IIIB) and Sup T1 cells in the presence of a variety of mutant D70A GRFT concentrations. Data represent the mean of at least two to three independent experiments. (TIF) [file pone.0064132.s003.tif]

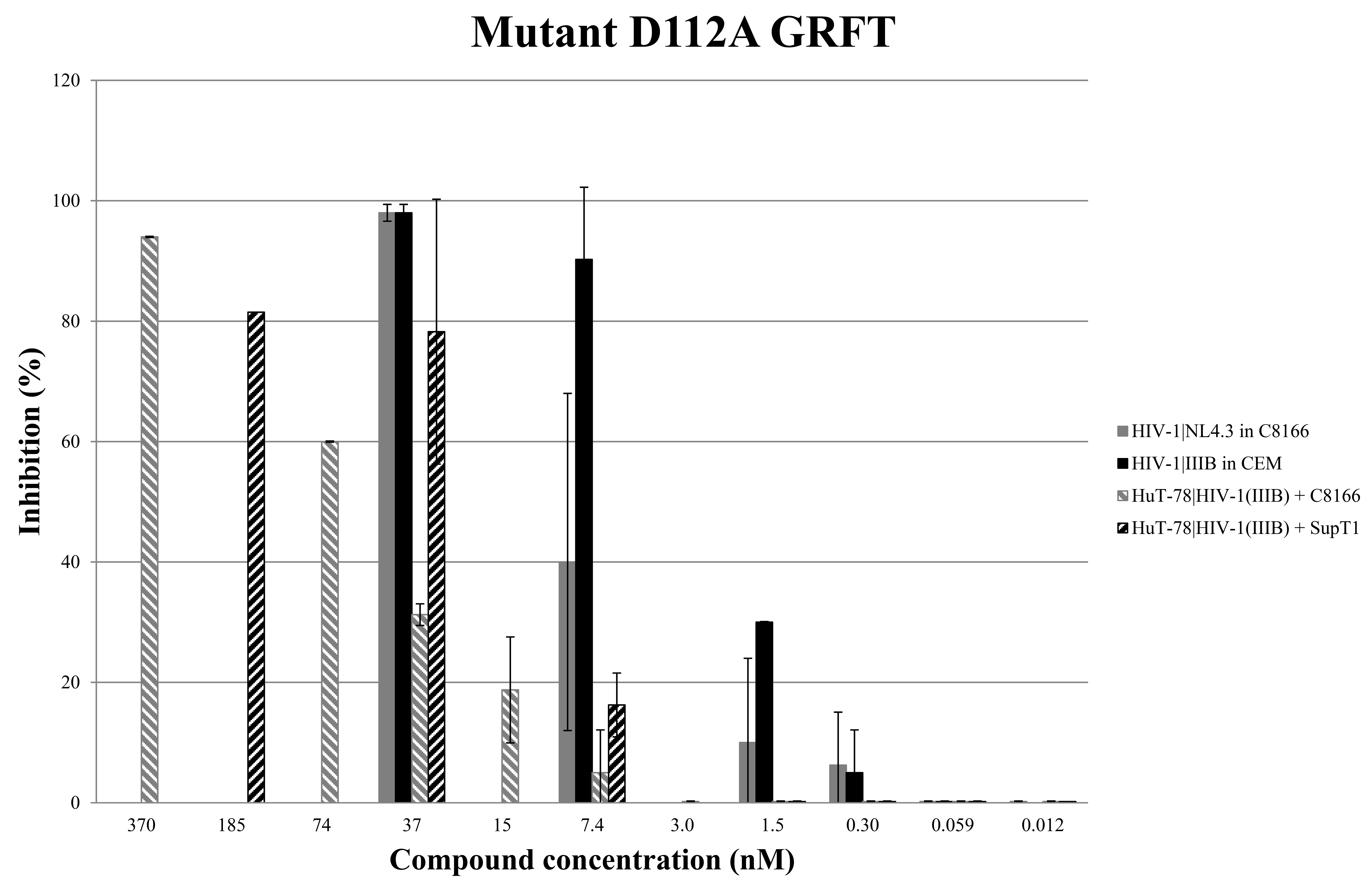

Supplement: Figure S4 — Inhibition of virus-induced cytopathicity (syncytia formation) in HIV-1/NL4.3-infected C8166 cell cultures, HIV-1(IIIB)-infected CEM cell cultures, cocultures of HUT-78/HIV-1(IIIB) and C8166 cells, and cocultures of HuT-78/HIV-1(IIIB) and Sup T1 cells in the presence of a variety of mutant D112A GRFT concentrations. Data represent the mean of at least two to three independent experiments. (TIF) [file pone.0064132.s004.tif]

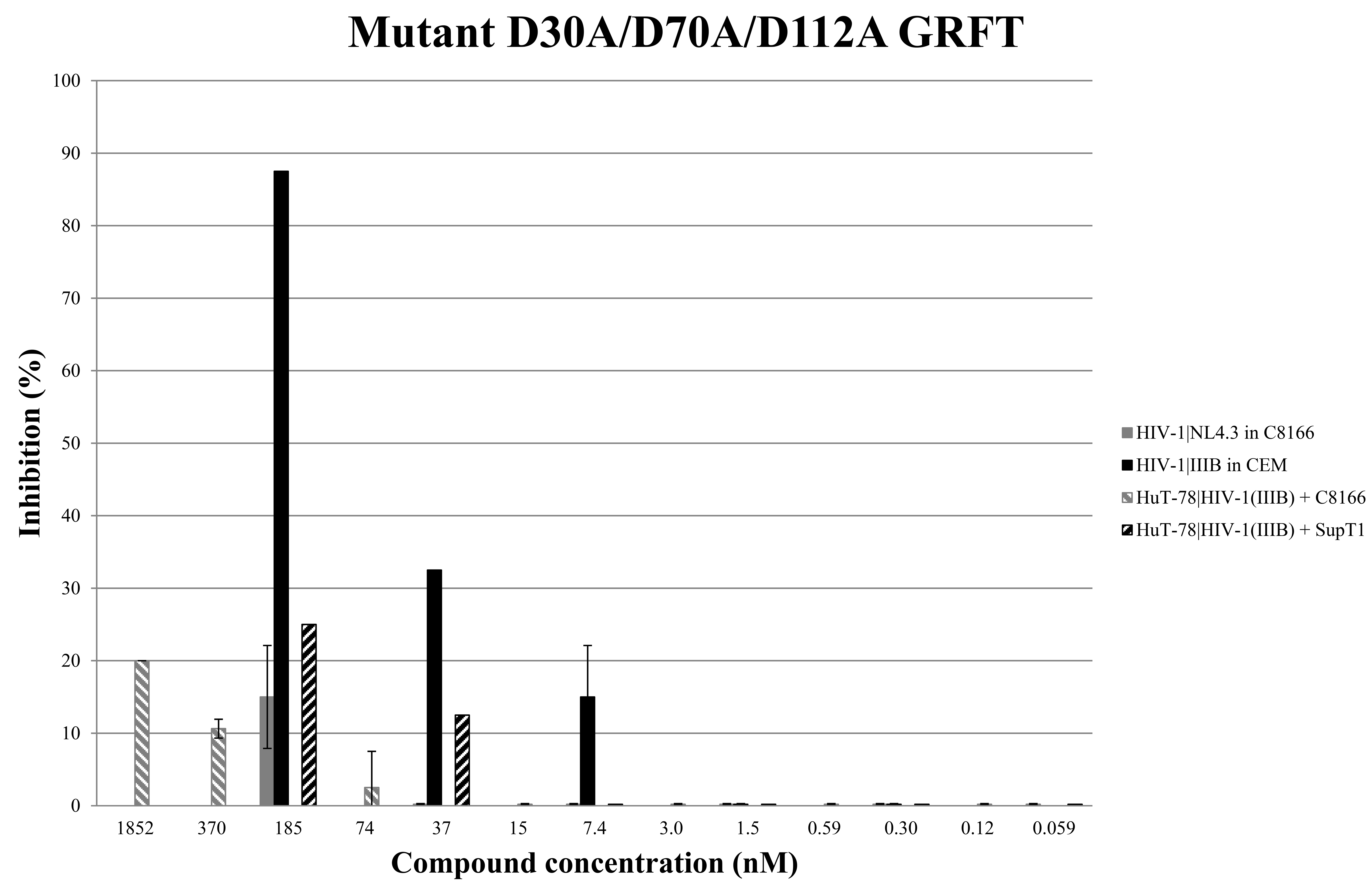

Supplement: Figure S5 — Inhibition of virus-induced cytopathicity (syncytia formation) in HIV-1/NL4.3-infected C8166 cell cultures, HIV-1(IIIB)-infected CEM cell cultures, cocultures of HUT-78/HIV-1(IIIB) and C8166 cells, and cocultures of HuT-78/HIV-1(IIIB) and Sup T1 cells in the presence of a variety of mutant Triple A GRFT concentrations. Data represent the mean of at least two to three independent experiments. (TIF) [file pone.0064132.s005.tif]

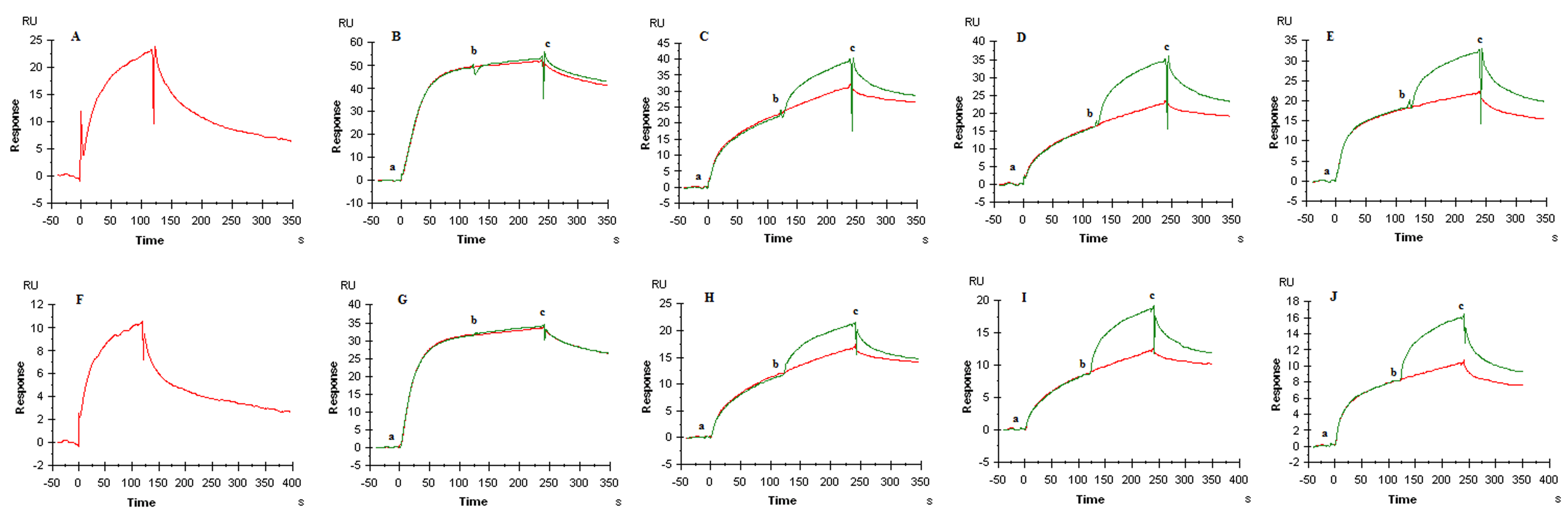

Supplement: Figure S6 — SPR-based competition experiment between CBAs and DC-SIGN for binding to immobilized gp120 ADA (chip density 130 RU ∼1.1 fmol) (Panels A–E) and gp120 IIIB (chip density 90 RU ∼0.8 fmol) (Panels F–J). Panel A: Injection of 200 nM DC-SIGN on gp120 ADA. Panel B: Effect of DC-SIGN exposure to HIV-1(ADA) gp120 that had been preexposed by WT GRFT. 5 nM WT GRFT was injected (time point a; red and green curves), followed after 2 min by an additional injection (time point b) of 5 nM WT GRFT (red curve) or WT GRFT +200 nM DC-SIGN (green curve). In Panel C till E similar experiments were carried out but 5 nM WT GRFT is replaced by 10 nM D30A GRFT; 20 nM D70A GRFT or 10 nM D112A GRFT, respectively. Data of similar experiments are shown in Panels F till J, using gp120IIIB as the covalently linked analyte. The curves show a representative example out of two independent experiments. (TIF) [file pone.0064132.s006.tif]
